# Developing a typology of mentoring programmes for young people attending secondary school in the United Kingdom using qualitative methods

**DOI:** 10.1016/j.childyouth.2018.03.025

**Published:** 2018-05

**Authors:** Heide Busse, Rona Campbell, Ruth Kipping

**Affiliations:** Bristol Medical School, Population Health Sciences, University of Bristol, Bristol, United Kingdom

**Keywords:** Mentoring program, Adolescent, Schools, Classification, United Kingdom, Qualitative research

## Abstract

Mentoring programmes are commonplace and delivered in a range of different ways in the United Kingdom and North America. To better understand the type of programmes available and to inform future evaluations, we developed a typology of formal mentoring programmes for young people in secondary schools in the United Kingdom. Telephone interviews with 23 programme managers from purposively sampled mentoring organisations were conducted and analysed using thematic and framework analysis. The typology was consulted on with five experts in mentoring. The final typology differentiates mentoring programmes by three overarching categories: type of mentor (older student, school staff, adult volunteer, paid adult), programme setting (school, community, online) and programme aim. The findings suggest that although mentoring programmes are heterogeneous, it is possible to group programmes into ‘personal and developmental’ and ‘academic and employability’ mentoring programmes and to differentiate between 12 overall mentoring models. The typology helps understand what is being delivered and how, which is a necessary precursor to any evaluation of health, educational, relational and social outcomes.

## Highlights

• Formal mentoring programmes for young people are heterogeneous.
• Programmes can be distinguished by taking account of the type of mentor, programme setting and aim.
• Programmes can be grouped into ‘personal and developmental’ and ‘academic and employability’ programmes.
• Twelve distinct models of youth mentoring programmes have been identified in the United Kingdom.

## List of abbreviations

**Table:** 

UK	United Kingdom
USA	United States of America
PDM	Personal and Developmental Mentoring
AEM	Academic and Employability Mentoring
BBBSA	Big Brothers Big Sisters of America

## 1 Introduction

An increasing number of young people experience psychological, social and behavioural difficulties in their transition to adulthood (Inchley et al., 2016; Patton et al., 2016) which can have deleterious consequences for the young people and society (Mokdad et al., 2016; Sawyer et al., 2012; Scott, Knapp, Henderson, & Maughan, 2001). A range of approaches, based upon different levels of the socio-ecological model of health (Dahlgren & Whitehead, 1991), are utilised to advance the health and wellbeing of young people. One individual-level intervention that is commonplace, popular and perceived by many to be effective is formal youth mentoring (Colley, 2003; Raposa, Dietz, & Rhodes, 2017; Rhodes & DuBois, 2008). Compared to informal ways of mentoring, formal mentoring programmes explicitly recognise the mentoring relationship and usually involve matching a selected young person (mentee) to another individual (mentor). The establishment of a close relationship between mentor and mentee, characterised by mutuality, trust and empathy, is hypothesised as key in leading to beneficial socio-emotional, cognitive and identity development (Rhodes, 2005).

Influenced by developments in North America, formal mentoring programmes have grown rapidly in the United Kingdom (UK) and now operate in various settings and contexts (Philip, 2003; Philip & Spratt, 2007; Social Exclusion Unit, 1999). Youth mentoring is an approach which was advocated in the recent Lancet Commission on adolescence (Patton et al., 2016) and it attracts support from national and local government (UK Government, 2017), from third sector organisations (Philip & Spratt, 2007) and is widely used in schools (Parsons et al., 2008).

Given the plethora of mentoring programmes in existence, it is important to assess whether formal mentoring programmes in the UK are effective and cost-effective in improving young people's health, wellbeing, educational, employment and training outcomes.

In the following sections, we review what formal mentoring programmes are, what is known about the effectiveness of mentoring programmes and previous ways that have been used to make sense of the multiplicity of what is considered as ‘mentoring’ programmes.

### 1.1 Definitions of formal mentoring programmes

As Freedman noted in 1991, “Mentoring is flexible, accommodating whatever attributes people want to give it” (Freedman, 1991, p. 37). Various definitions have been proposed in previous research (DuBois & Karcher, 2005) with no commonly used definition of youth mentoring in either research or practice (Stewart & Openshaw, 2014). Definitions generally focus on three core elements: (i) the mentor being someone with greater experience than the mentee, (ii) the mentor offering guidance or instruction with the intent of facilitating the mentee's growth and development, and (iii) the fact that there is an emotional bond between mentor and mentee (DuBois & Karcher, 2005). Regardless of definition, the centrality of the establishment of a trusting and supportive relationship between mentor and mentee is key in most programmes that are referred to as mentoring programmes (Rhodes, 2005).

Given this broad and flexible definition, there is a range of different types of programmes that are referred to as ‘mentoring’ and that these programmes can vary widely (Karcher, Kuperminc, Portwood, Sipe, & Taylor, 2006; Raposa et al., 2017). The Big Brothers Big Sisters of America (BBBSA) programme is arguably the most well-known mentoring programme, currently operating across all States in America and in 14 other countries (Big Brothers Big Sisters International, 2014; Stewart & Openshaw, 2014). BBBSA typically involves matching a young person with an unrelated adult volunteer mentor to engage in regular meetings for a minimum of one year duration (Big Brothers Big Sisters International, 2014). Whereas historically programmes consisted of one-to-one mentoring approaches and in-person meetings using adult volunteers alike the BBBSA model, programmes and approaches to youth mentoring have extended to group mentoring (Jent & Niec, 2009), programmes using online mentoring (Rhodes, Spencer, Saito, & Sipe, 2006), and programmes working with paid, professional mentors (Eddy et al., 2017). In addition to differences between programmes in their formats and type of mentor used, programmes also differ in other characteristics. For example, each BBBSA agency has their own requirements regarding the frequency of meeting and length of each meeting between mentor and mentee (Tierney, 1995).

### 1.2 Evidence of effectiveness of formal youth mentoring programmes

The earliest robust evaluation of a formal mentoring programme, employing a randomised control trial design, concerned the BBBSA community-based programme which paired young people from typically single-parent households with a mentor (Grossman & Tierney, 1998; Tierney, 1995). The evaluation involved 1138 young people aged 10–16 years and highlighted that young people that received a mentor, compared to the wait-list control group, showed lower drug and alcohol use, improved attendance and performance at school, improved relationships with parents and peers and less fighting at the 18 months follow-up (Grossman & Tierney, 1998; Tierney, Grossman, & Resch, 1995). No impact was reported on feelings of self-worth, self-confidence or social acceptance (Tierney et al., 1995). A few years later, the BBBSA school-based programme was evaluated and highlighted that those who received a mentor achieved better educational outcomes and reported more positive perceptions of their academic abilities at the end of the school year, but there was no strong statistical evidence with regard to relationships with others or problem behaviours (Herrera, Grossman, Kauh, Feldman, & McMaken, 2007; Herrera, Grossman, Kauh, & McMaken, 2011).

When looking at mentoring programmes in the UK, a few individual programmes have been subject to robust evaluation, with no randomised control trial yet undertaken of a mentoring programme for young people of secondary school age. Past evaluations include a national evaluation of youth justice board mentoring schemes for young people who already had or were at risk of offending. This evaluation concluded that programmes resulted in re-enrolling approximately 45% participating young people back into education or training (St James-Roberts, Greenlaw, Simon, & Hurry, 2005). Another evaluation was conducted of the ‘Mentoring Plus’ programme revealing that the programme generally led to increased engagement in education, training and work but reported that no clear evidence was found on offending behaviour, family relationships, substance misuse and self-esteem (Shiner & Barriers, 2004). The evaluation of the London Major's mentoring scheme reported improved school and academic outcomes for young people with a mentor, however, the evaluation also revealed that particularly youth with high needs were less likely to sustain the mentoring relationship, highlighting that programme outcomes might have differed for the individual young people (Greater London Authority, 2015). It has to be noted that evaluations of UK mentoring programmes mainly used qualitative methods such as case studies, without control groups and are therefore limited in their design.

A range of systematic reviews have been undertaken to scrutinise the available evidence on mentoring with regard to a range of different outcomes including academic, health, relational, and social outcomes of young people. Whereas only some reviews focussed solely on randomised control trial evaluations (Thomas, Lorenzetti, & Spragins, 2011; Thomas, Lorenzetti, & Spragins, 2013a; Thomas, Lorenzetti, & Spragins, 2013b), others included a variety of study designs. The majority of reviews were based on programmes in the United States of America (USA) where most studies have been undertaken. In their review of 73 youth mentoring programmes, involving 83 independent research samples, DuBois and colleagues revealed modest effect sizes across emotional, behavioural and educational domains (DuBois, Portillo, Rhodes, Silverthorn, & Valentine, 2011). Meta-analyses have revealed that compared to non-mentored individuals, mentored individuals were more likely to perform better (Eby, Allen, Evans, Ng, & DuBois, 2008), report positive interpersonal relationships (DuBois et al., 2011; Eby et al., 2008)and were found less likely to engage in delinquency (Tolan et al., 2013) and present with conduct problems (DuBois et al., 2011) or withdrawal behaviours (Eby et al., 2008). Reviews of school-based mentoring programmes have concluded that mentoring might be able to positively influence a young person's relationships and connectedness to others (Wood & Mayo-Wilson, 2012) and improve self-esteem (Randolph & Johnson, 2008) and educational outcomes such as school attendance, academic achievement and attitudes towards school (DuBois et al., 2011; Eby et al., 2008; Tolan et al., 2013).

Despite some promising findings, not all reviews reported evidence of statistical significance in the outcome domains and effect sizes reported have generally been described as moderate or small (Eby et al., 2008; Rhodes & DuBois, 2008) and some may have been due to chance. For instance, no statistically significant effects was found in the health domain in DuBois's systematic review (DuBois et al., 2011) nor was any effect of mentoring observed on academic attitude, achievement and attendance in Randolph's review of school-based programmes (Randolph & Johnson, 2008). Meta-analyses have reported no statistical significant effect of mentoring on young people's evaluation of psychological stress and strain (Eby et al., 2008), motivation or involvement (Eby et al., 2008), helping others (Eby et al., 2008), measures of aggression (Tolan et al., 2013), smoking (Thomas et al., 2013b) and there have been mixed findings with regard to young person's drug use or alcohol use (Thomas et al., 2011; Thomas et al., 2013a).

When trying to make sense of the available evidence, it is important to consider that systematic reviews differ in the type of studies that they included as ‘mentoring programmes’. This might in part be due to the lack of a unifying definition of mentoring or mentoring programmes [18]. For instance, DuBois's systematic review explicitly stated that the definition of mentoring was kept broad and that some programmes “fell at the conceptual boundaries of traditional conceptualisations of youth mentoring” (DuBois et al., 2011, p. 67). In their review, authors therefore grouped a range of different programmes together to capture the evidence of effectiveness including programmes providing group mentoring (Jent & Niec, 2009), a one-to-one mentoring programme to delay second births among adolescent mothers (Black et al., 2006) and a programme aiming to prevent obesity and promote healthy behaviours in adolescence by matching youths with college students training in motivational interviewing techniques (Black et al., 2010). Each of the included studies had individual programme features and components, used different forms of mentoring, and used mentoring for different target groups. Due to the multiplicity of different programmes whose effect sizes are combined, findings from systematic reviews must be assessed with a note of caution and only provide limited insight into which type of mentoring programmes work, for whom and why. Identifying the components of mentoring programmes is critical as programme-related practices have been established to be able to influence the effectiveness of mentoring and consequently youth outcomes (Rhodes & DuBois, 2008).

For example, one factor that has been established to impact on the effectiveness of a programme is the overall length of the relationship between mentor and mentee, also referred to as match length. Research based upon the BBBSA evaluation revealed that the impact of mentoring on outcomes of the young people with a mentor become increasingly stronger with a longer match length (Grossman & Rhodes, 2002). In fact, young people who had a short duration of mentoring or where the mentoring was ended prematurely reported a decrease in their global self-worth and perceived academic competence (Grossman & Rhodes, 2002). In line with these findings, other studies have reported that early determination of the mentoring relationship or short length of mentoring have been associated with negative outcomes (DuBois, Holloway, Valentine, & Cooper, 2002; Spencer, 2006), however, it has also been emphasised that this is a common experience of many mentees (Grossman & Rhodes, 2002; Spencer, Basualdo-Delmonico, Walsh, & Drew, 2017). When looking at existing programmes and those included within existing systematic reviews, their overall durations differ widely (DuBois et al., 2011), therefore possibly impacting on assessments of overall evidence of effectiveness.

The lack of robust evidence on youth mentoring's effectiveness coupled with the fact that most of the extant evidence is for programmes in the USA (DuBois et al., 2011; Eby et al., 2008), means there is an urgent need to evaluate formal youth mentoring programmes in the United Kingdom.

### 1.3 Towards a typology of formal mentoring programmes

In order to evaluate the effectiveness of any intervention, a clear and detailed understanding of the intervention and intervention context is required (Bonell, Fletcher, Morton, Lorenc, & Moore, 2012; Craig et al., 2008). Among other reasons, this is important so that the intervention is replicable if found to be effective. A plethora of what is referred to as ‘mentoring’ programmes exist in the UK, with various different aims and objectives, underlying structures and formats (Colley, 2003; Philip & Spratt, 2007). Before any wide-ranging evaluation can take place, it is necessary to investigate programme's similarities and differences and to develop a classificatory system for formal mentoring programmes. This is particularly important as an evaluation may require the inclusion of multiple similar programmes to provide an adequate sample size for the evaluation.

The aim of a good classification is to (i) identify the key dimensions that define the topic of interest and (ii) to order entities of the topic of interest into one or more mutually exclusive and independent categories (Bailey, 1994). Compared to taxonomies which use hierarchical dimensions, typologies use non-hierarchical dimensions and are well suited to the task of classifying ill-defined concepts (Bailey, 1994; Petticrew & Roberts, 2003; Ritchie, Lewis, Nicholls, & Ormston, 2013). This is why a typological approach to the classification of formal mentoring programmes was deemed appropriate for this study.

Multiple studies have attempted to form typologies in the field of mentoring, for instance, to differentiate between types of relationships between mentor and mentee (Langhout, Rhodes, & Osborne, 2004; Pryce & Keller, 2007; Simon & Eby, 2003) different types of mentors (Ford, 1998; Scanlon, 2009) or to summarise negative mentoring experiences (Simon & Eby, 2003). Classifying different types of mentoring relationships was the aim of Karcher and Nakkula's work, who distinguished mentoring relationships by looking at the focus, the purpose and the authorship and what is termed the ‘developmental’ and ‘instrumental’ style of relationships (Karcher & Nakkula, 2010).

A few other typologies have been developed to differentiate mentoring programmes from one another (Eby, 1997; Karcher et al., 2006; Saito & Blyth, 1992; Scanlon, 2009; Sipe & Rogers, 1999). However all were based on programmes in the USA and were typically broad in their scope, encompassing a wide age range and informal and formal mentoring strategies. Three typologies of aspects of mentoring in the UK have been published (Ford, 1998;Philip & Hendry, 1996; Philip & Spratt, 2007).

Based on interviews with young people in Scotland, Philip and Hendry (1996) identified five common mentoring styles, referred to as ‘classic’, the ‘individual/team’, the ‘best friend’, the ‘peer group’, and the ‘long term risky adult’. Philip and Spratt (2007) further differentiated between five overall aims which they classified as ‘compensatory’, ‘instrumental’, ‘expanding opportunities’, ‘reduction of unwanted behaviour’ and ‘integration into community’ based upon a literature review. Ford (1998) described different mentoring styles utilised by career guidance mentors but it is unknown how these styles were derived. As with other attempts at the classification of mentoring, the categories used were not mutually exclusive (Philip & Hendry, 1996; Philip & Spratt, 2007).

Summarising the attempts to conceptualise mentoring through typologies, Hall (Hall, and Scottish Council for Research in Education, E, 2003) concluded that most classifications or models draw on the following four overall dimensions in order to categorise mentoring programmes: (i) the origin of mentoring (natural vs. formal mentoring), (ii) their purpose (instrumental vs expressive), (iii) their format (one-to one or group), and (iv) the site of the mentoring (site-based or community-based).

It is important to note that these dimensions were derived from reviewing past classifications, which in turn were mainly developed based on programmes in the USA. Given the individual nature of mentoring programmes and contexts in which mentoring programmes operate in, it is uncertain how mentoring programmes in the UK can be best classified.

### 1.4 Current study

Contrary to many existing classifications, we were interested in deriving a typology that used mutually-exclusive categories, to be able to find a way of grouping programmes to not only aid the future evaluation of different programme models, but also to disentangle the effects that different mentoring programmes might have. Providing a framework for separating different mentoring programmes from one another might also guide future research on the effectiveness of certain groups of programmes. Past research has highlighted that mentoring programmes differ widely and that by grouping different programmes together, their possible effect size might be undermined or inaccurate.

As past attempts at classifications of mentoring have largely been based on mentoring relationship characteristics, types of mentors, USA-based programmes, informal and formal mentoring practices, and on broader target groups, the purpose of this study was to develop a typology of currently active mentoring programmes that are offered and available for young people in secondary schools in the UK (England, Scotland, Wales and Northern Ireland).

We were interested in young people within secondary schools as adolescence is regarded as a crucial time in which trajectories towards health and wellbeing in adult life can be modified (Viner et al., 2012). We were interested in looking at a broad target population, rather than at young people with very specific characteristics (e.g young people in care) as we are concerned with identifying the types of programmes that have the potential to be expanded for use for a broad range of young people.

From what is known about programmes in the United Kingdom, we expected there to be a variety of different programmes in existence, both within and outside of schools. We did not have a prior hypothesis about how we would group programmes.

## 2 Methods

### 2.1 Eligibility criteria

Mentoring organisations were eligible for inclusion in the study if they provided one or more formal mentoring programmes to young people enrolled in secondary schools in the UK. Young people were assumed to be 11–16 years old which is equivalent to Years 7–11 within English secondary schools. To capture the range of potential organisations and programmes, the definition of mentoring used in the study was kept broad and inclusive. Mentoring was defined as ‘any programme between an identified young person and other individual(s), aimed at the support of the young person’ and that is referred to by the provider organisation as ‘mentoring’. Mentoring organisations that provided mentoring programmes solely for another group of the population (e.g. primary schools) or that were not operating in the UK were not eligible for inclusion.

### 2.2 Identification of mentoring programmes

A pilot search established that most organisations listed as part of national mentoring networks websites had an internet presence. Consequently, a website search was undertaken to identify UK organisations that provided formal mentoring programmes for young people. The search contained three elements: (i) search of two national mentoring network websites operating in England and Scotland; (ii) search of relevant charitable organisation and trust registers; and (iii) a Google search to identify programmes in Wales and Northern Ireland where no mentoring networks were known to exist. Details of websites searched and search results can be found in Appendix A. A total of 815 organisations were identified and information on these organisations and their mentoring programmes were captured in an Excel spreadsheet. Through looking at the available information of each programme, we identified 163 organisations that met the eligibility criteria for our study. To ensure the development of a comprehensive typology, maximum variation sampling based on country (Wales/England/Scotland/Northern Ireland) and the type of mentoring programme (by setting, provider organisation and format of programme) was used to recruit a purposeful sample of organisations.

### 2.3 Participants

Programme managers of selected organisations were invited to take part in semi-structured telephone interviews to obtain a thorough account of their mentoring programme(s) and to explore views on how mentoring programmes differ. Programme managers were selected as they were regarded as having insight into the details of their mentoring programme(s), programme history and wider experiences of delivering and managing mentoring programmes.

Experts in the field of mentoring in the UK were invited to take part in telephone interviews to provide their views on the draft typology. Academic and practitioner experts were identified through the literature and consultation with other researchers. An important criterion in the selection process was that these experts were not involved in the interviews used to draft the typology.

### 2.4 Ethical approval

All participants provided written consent and ethics approval was granted by the University of Bristol Faculty of Health Sciences Research Ethics Committee in 2015 (reference number 24341). Given the relatively small numbers of what can be considered mentoring ‘experts’ in the UK, experts in this study were asked for their consent to be acknowledged by name.

### 2.5 Procedures

**Fig. 1:**
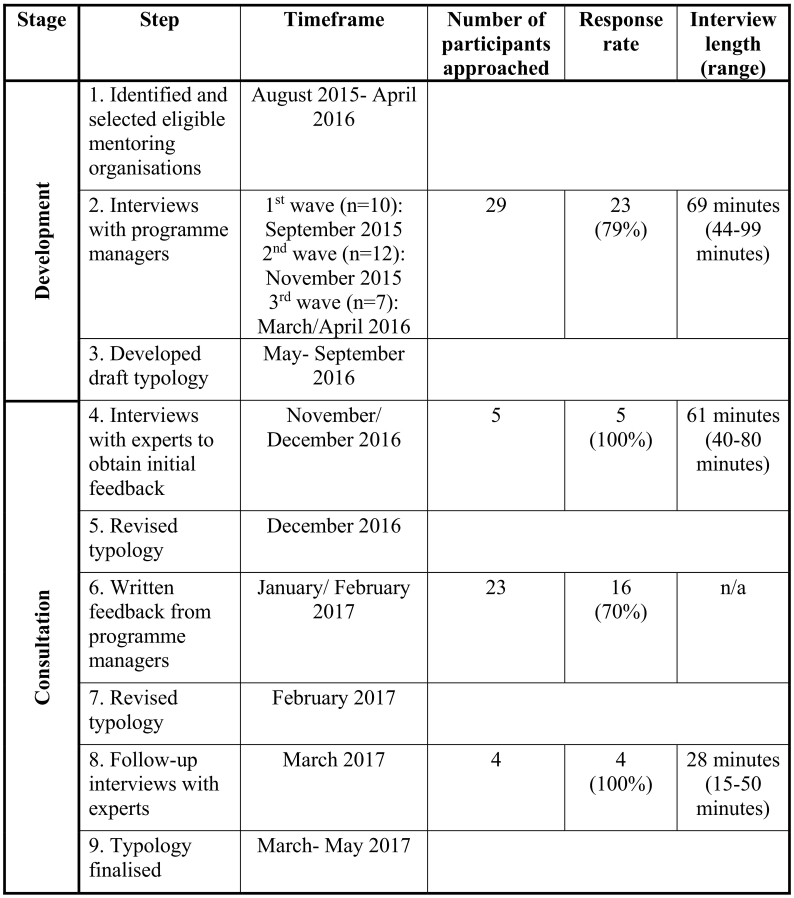
Overview of participants and steps of research.

The research was carried out in two main stages as displayed in Fig. 1. The first stage involved programme managers and aimed to develop a draft typology (Step 1–3). The second ‘consultation’ stage involved experts and programme managers and its purpose was to revise and finalise the typology (Step 4–9). Data collection was conducted concurrently with data analysis and continued until data saturation was reached. Thus data collection ceased at the point at which no new variation in the way in which mentoring was provided emerged in interviews. This happened during the third wave of interviews when the decision was made to progress drafting the typology.

Programme managers were asked in the interview (Step 2) whether they would like to take part in the consultation on the draft typology (Step 6). During the first interview (Step 4), experts were asked whether they would be interested in taking part in a second, follow-up interview (Step 8). As part of the consultation process, all participants were assured that that there were no right or wrong answers and were encouraged to critique the draft typology.

All telephone interviews were facilitated using a flexible and semi-structured topic guide and audio-recorded using a digital password encrypted audio recorder (Olympus DS-3400). Interview recordings were transcribed verbatim and anonymised. Data were stored on a secure, password protected, electronic database.

### 2.6 Data analysis

Anonymised transcripts were read and re-read and analysed thematically (Braun & Clarke, 2006). A selection of transcripts was shared with two other members of the team who were not involved in the data collection. Researchers met to discuss the emerging themes and characteristics for the typology development. Where there was disagreement, original interview data were scrutinised against the coding and discussed until agreement was reached. Codes were created and compared using constant comparison derived from grounded theory (Charmaz & Belgrave, 2002). Based on the codes, a first initial conceptual framework was produced. This was further refined and adapted as the data analysis progressed. The final framework was re-created in NVivo10 to simplify the process of assigning data to the relevant codes.

Additional to the inductive approach to the data analysis, the framework approach was employed to aid the typology development (Gale, Heath, Cameron, Rashid, & Redwood, 2013; Ritchie et al., 2013). A central question asked in each interview concerned perceived differences between formal mentoring programmes in the UK. Answers were taken as a starting point from which to distinguish between mentoring programmes within the typology. A matrix was created based on all identified categories, the ‘codes’ presented in columns and all formal mentoring programmes, the ‘cases’, presented in rows within a Microsoft Excel spreadsheet. Working inductively with the data to derive categories for the typology and then applying these deductively ensured that the typology was nested within the data (Morse, Barrett, Mayan, Olson, & Spiers, 2002). The draft typology was then discussed by the authors and amended based on the feedback received in the consultation process. All researchers agreed on the final emerging themes and categories used in the typology.

## 3 Results

Twenty-three programme managers and five experts took part in the research. An overview of participants and the stages of research is presented in Fig. 1. Quotations from programme managers have the indication 'M' and experts the indication 'E'.

### 3.1 Description of participants

Programme managers had various career backgrounds; most had previously worked with young people for example as learning mentors, or in a sports or youth work context. Participants varied in the amount time that they had been working for their respective organisations (from a few months to 17 years). Most had worked for the organisation for more than five years.

The participants who were experts in the consultation had various job roles and were either practitioners or academics. Most experts indicated that they had been involved in the mentoring field for over 10 years. To our knowledge, most of the experts were based in the UK when the research was undertaken.

### 3.2 Description of mentoring organisations and programmes

Of the 23 managers that took part, 21 were involved in organisations that provided and delivered one or more formal mentoring programmes to secondary school students. One interviewee worked in an organisation that provided informal mentoring and one respondent worked in an organisation that provided online mentoring infrastructure. Two thirds of the organisations provided mentoring as part of a wider service for young people, involving non-mentoring services. Whereas most organisations delivered the mentoring programme themselves, two organisations commissioned another organisation to deliver the programme.

A total of 35 formal mentoring programmes were described by participants of which 28 were provided for UK secondary schools students and described in sufficient detail to be included in the analysis. An overview of the characteristics of mentoring organisations and programmes is presented in Table 1.

**Table 1:** Characteristics of mentoring organisations and programmes.

	Category	Subcategory	Number	Percentage
Characteristics of mentoring organisations (*n* = 23)	Country of operation of organisation	England	14	61%
		Wales	3	13%
		Scotland	3	13%
		Northern Ireland	2	9%
		Multiple countries	1	4%
	Location	Single city	11	48%
		Multiple cities	2	9%
		Single region	5	22%
		Multiple regions	3	13%
		Nationwide	2	9%
	Number of young people supported by organisation each year	<25	2	9%
		25–50	3	13%
		51–100	3	13%
		101–500	8	35%
		501–1000	2	9%
		1001–5000	2	9%
		>5000	1	4%
	Funder of organisation	Secondary schools	5	22%
		City council	1	4%
		University	2	9%
		Education Funding Council	1	4%
		Mixture of funding	14	61%
	Years organisation provided mentoring	<3 years	1	4%
		3–5 years	4	17%
		6–10 years	7	30%
		11–15 years	5	22%
		>16 years	4	17%
	Provider of formal mentoring programme^a^	Mentoring organisation	10	48%
		Youth or youth service organisation	3	14%
		Secondary school	4	19%
		University	2	9%
		Partnership Federations	2	9%
Characteristics of mentoring programmes (*n* = 28)	Method of delivery	Face-to-face only	25	89%
		Online only	1	4%
		Combination of face-to-face and online mentoring	2	7%
	Adult or peer mentor	Adult mentor	24	86%
		Peer mentor	4	14%
	Number of mentors given to mentee	One mentor	20	71%
		Multiple mentors	8	29%
	Overall duration	< 5 months	8	29%
		6–11 months	5	18%
		12 months	8	29%
		>12 months	1	4%
		Not known^b^	5	18%
	Frequency of meetings	Multiple times per week	1	4%
		Weekly	14	50%
		Bi-weekly	5	18%
		Once a month	3	11%
		Not known^b^	5	18%
	Length of each meeting	About 30 min	6	21%
		About 45 min	1	4%
		1 h	4	14%
		Between 1 and 2 h	2	7%
		2 h	5	18%
		>2 h	1	4%
		Not known^b^	7	25%
	Timing	During school time	17	61%
		After school time	9	32%
		Not known^b^	2	7%
	Predominant setting	School	18	64%
		Community	8	29%
		No setting (online only)	2	7%
	Is a formal match undertaken between mentor and mentee?	Yes	21	75%
		No	5	18%
		Not known^b^	2	7%
	Mentoring on its own or in combination with other activities	Mentoring alone	9	32%
		Mentoring and other programme component	12	43%
		Not known^b^	7	25%
	Funding	Internally-funded	11	39%
		Externally-funded	17	61%

a Based on all organisations/institutions that provide one or more formal mentoring programmes (*n* = 21).

b This information was not discussed in the interview regarding the particular programme.

Whereas some internally-run school-based programmes were open to all young people, most externally-run programmes referred to a set of eligibility criteria that either the schools or individual young people had to meet to access the programme. These criteria included school-level characteristics (e.g. percentage of free school meals, locality, and performance ratings) and individual-level characteristics (e.g. socio-economic status, at risk of not being in education, employment or training). Most programmes worked with mentors of a range of ages, ethnicities, gender and professional backgrounds who typically acted in a voluntary capacity.

### 3.3 Typology development

Participants were asked about differing features between mentoring programmes provided to secondary school students in the UK. Interviewees acknowledged the multiplicity of formal mentoring programmes in existence and gave insight into a few different programme types underlying the fact that mentoring programmes take many different forms.

“It's an alphabet soup, there are hundreds of them [mentoring programmes].”

(Manager M5)

“I mean they [mentoring programmes] differ dramatically in terms of aims and objectives, mentoring can be applied for so many different things.”

(Manager M13)

“And then, one-to-one mentoring which happens completely independently almost, where you might meet up in a coffee shop, discuss things. There is mentoring groups who are going into schools along like tutors, […] I think, it's, it's evolving constantly and it's taking a lot of different forms.”

(Manager M12)

Participants spoke at length about differences in mentoring programmes and mentioned many ways in which they thought programmes differed from one another. Two participants expressed feeling unsure about other mentoring programmes and this was not further discussed in the interview. A total of 20 categories which distinguished between programmes were explicitly mentioned by participants: an additional 13 were identified in the data analysis. A list of all 33 categories is presented in the Appendix B and a brief description of each category is given in Appendix C.

Categories included those capturing information about the mentoring organisation (i.e. type of provider, size, location, type of funding received), characteristics of mentor and mentee (i.e. remuneration of mentor, whether participation is voluntary or not), mentoring relationship details (i.e. whether or not a formal matching process is undertaken), programme delivery (i.e. setting, method of delivery, format, time of delivery, intensity, frequency, overall duration) and details of mentoring sessions (i.e. who is present in sessions, whether the session is structured or unstructured).

Each of the categories and the 28 mentoring programmes were examined for general patterns or programme models that were able to classify mentoring programmes into more than one exclusive category. Generally, mentoring programmes were provided in a variety of different delivery pathways and settings and no two mentoring programmes were described in the same way. Programmes typically overlapped on a few of the categories but differed on other categories. For instance, so called peer-mentoring programmes delivered within schools were similar in that they had they utilised the same setting and the same type of mentor (older students from within the same school), but they were used for different purposes. Whereas some programmes focussed on facilitating the transition to and from secondary school, other programmes focussed on older students helping the younger ones in providing support to increase academic skills and results. Other differences persisted regarding the overall duration of the programme, how regularly mentor and mentee would meet, etc.

Despite their heterogeneity, analysis of the data found that programmes can be best distinguished from one another by looking at the following three over-arching categories: the setting, the type of mentor(s) and the programme's overall aim. These factors clearly distinguished between programmes and were seen to impact on a variety of other programme components such as its delivery, underlying assumptions and expectations. Each of these categories with supporting quotations from participants will be described in turn.

(i) Programme's overall aim

The foremost category used to distinguish between programmes was the aim of programmes. This was also referred to as the focus or intended outcome of the mentoring programme and was the category that was mentioned the most.

“I personally think that one of the best ways to differentiate is by outcome. So what is it you are trying to achieve with your mentoring programme?”

(Manager M23)

Aims of mentoring programmes were regarded to impact on the overall process of mentoring from the start, in the recruitment and training of mentors, to the end as they influenced the types of outcomes that a mentoring programmes aims at and uses to assess its potential impact. Furthermore, the aim of mentoring was seen to impact on the basis of the conversation, communication and generally on what goes on in the mentoring sessions between mentor and mentee as described by the following participant:

“Because we come from a kind of employability and professional friend type scenario, we always bringing it around to the kind of jobs and the kind of careers and the courses that they should be looking at whereas some schemes will just be talking about the personal issues, the, the problems, the home life, you know, that kind of thing.”

(Manager M22)

As one interviewee stated, the type of mentoring provided also corresponded to the funding that is sought and obtained.

“When we are looking at funding that people go for different types of mentoring, so there is that, you know, the social, kind of socially disadvantaged route or, you know, real problems route, and we don't really, we don't do that. We don't focus on that. So I think those are the two main areas of differences.”

(Manager M22)

Whereas some programmes aimed to improve and help the current situations a young person was in (i.e. help with learning and academic achievement in school), other programmes were aimed towards supporting a young person's future choices (i.e. help to plan career and university options). Furthermore, programmes differ with regard to the focus of their aim, whether this was quite specific (i.e. help with GCSE learning) or rather broad (i.e. to help develop life skills). One participant, part of an organisation that provided multiple mentoring programmes, gave insight into the way a distinction was made between programmes.

“How do they differ? I suppose that, that the way that we kind of, the way that we, I, split it up in here I think is probably the clearest distinction. Where you have got academic, where the results are focussed on the academic and underachievement and trying to push people on in terms of their grade and their performance in tests and their learning, improving their learning, to that more, I suppose, dealing with kind of social issues, and dealing with issues of a more, of a more personal nature, personal development issues […] that has to do with relationships and bullying and, kind of, just things that are affecting the young people in terms of their lives outside of the classroom. I think that's probably the clearest distinction that I have been able to make.”

(Manager M19)

(ii) Type of setting

The category that was further able to distinguish between programmes was the predominant setting where the mentoring programme was taking place, i.e. whether mentoring was provided within the school context, in the local community or predominantly online.

“The last thing is, you know, where does it actually take place? Do you go to the kids or do the kids come to you?”

(Manager M3)

This indicated the context in which the mentoring occurs which was regarded to determine the boundaries, capabilities and expectancies of programmes. For example, whereas mentoring within the school context was regarded as bounded by the school timetabling, safeguarding regulations, institutional rules and regulations and practical considerations like available spaces and facilities, mentoring offered in the community was seen to be more flexible and less bounded.

“It depends on the facilities in school. And they do vary quite a bit. So some of the schools corner off part of the learning centre and then they got the access to like computer and things, and the use of the bigger classrooms for three at the time. I think they basically have got those community rooms, but it is never sort of like rooms in isolation.”

(Manager M7)

Regarding mentoring programmes taking place within schools, interviewees highlighted that, for safeguarding as well as practical reasons, mentors worked in spaces such as the canteen, learning centres or open offices and emphasised the ‘private but public’ nature of the setting. This was contrary to many community programmes which were less closely monitored and where mentor and mentee sometimes met in isolated areas.

“They meet in, in what's called our [name for learning centre] […] But there are different, sort of little areas within there, where you can have some privacy because obviously in terms of confidentiality they have to be, and safety, they have to be in a place where they can be seen but not overheard. And a lady who works with us […] she is always in there when the meetings are taking place.”

(Manager M19)

“The majority of our schemes do meet at school, they would meet their student in reception area, they would then go to, a library or the school canteen or if they did use an office, it would be a kind of public office so, you know, have a door open, that kind of thing. So, that's the usual way it works […] it's in a private but public place.”

(Manager M22)

The setting also influenced the type of activities that were undertaken as part of mentoring. Whereas community-based programmes typically focussed on engagement in specific activities, such as sports, arts and crafts, most school-based programmes took place in a classroom setting and involved the mentor and mentee sitting down and talking together.

“Yes, it's out in the community. And it could take the form of any sort of activity that you can imagine. So, that could be as simple as going out for coffee, or it could be, going out for dinner, going to the cinema, horse-riding, kayaking, quad-biking, the list is endless. And, and it's all focussed around, initially anyway focussed around trying new experiences, and building a relationship of the back of that.”

(Manager M9)

Participants described processes by which mentoring in the community was monitored and procedures to make sure that mentoring was in line with safeguarding policies by undertaking risk assessments for mentors working one-to-one with a young person.

“So mentors always will text in and out to us, you know, just like they are a social worker or something, if they do a house visit, then they always need to text the management […] so, say they wanted to go to an isolated beach, I would know the area, I'd do a risk assessment for it, and then, you know, they would clearly let me know the times and they also let the parent know the times.”

(Manager M14)

The setting of mentoring also impacted on the timing of the mentoring session. Mentoring in the community typically happened after school or on weekends, whereas mentoring within the school would happen as part of school, sometimes provided within school lessons or at lunch times. Views differed on whether mentoring should happen in school time or outside of school and that this would influence the type of young people that were going to engage with the programme.

“They would meet outside of the school environment. Particularly- again, some of these young people have challenging relationships within schools to then to reinforce that with the mentor, we don't see it as a positive. So they would normally happen out of school days, so after 3 o'clock, early evening, night time, and part of that is also to engage the young person with positive activities in the local communities that they don't normally have access to so youth clubs, gyms, other activities, groups that they would not normally have the opportunity to go.”

(Manager M2)

“It's not that the school environment is a better setting, it is that you are very unlikely to persuade the at-risk young people to give up their afternoon or evening to go voluntarily in- to travel to go and see someone and be mentored. You wanna- you wanna work with the hard kids, yeah? You gonna need to go to them. They are not going to come to you.”

(Manager M3)

(iii) Type of mentor

Another category that was clearly able to distinguish between programmes was the type of mentor that provided the mentoring and whether this was an older student, a (paid) school staff member, an adult volunteer or paid adult. Whereas adult volunteers were usually seen to differ in characteristics from the young person that took part in the programme, paid adults were seen to sometimes be of similar characteristics, having experienced similar challenges as those taking part in the programme.

“We are not advisers through experience. So generally, many of the things we talk to young people about, we haven't experienced ourselves.”

(Manager M17)

Benefits and limitations were perceived with paying mentors for their work. It was acknowledged that whereas paying mentors for mentoring might attract different kind of mentors to the programme, the young people appreciated being mentored by individuals who voluntarily chose to spend their time with them.

“And particularly when I speak to the young people and I offer them this programme, I explain that its voluntary on their part, but I also make a point of telling them that the mentors, that I would be, you know, be matching them with, are volunteers, you know? They are not getting paid to spend time with you, they want to spend time with you, because they feel like they can make a difference. And that's always really received really well, because they, you know, especially the young people who are in, in the looked after care system, they constantly deal with changing faces, they constantly deal with people who are paid to do a job, and I think […] they relate really well to someone who is actually, you know, they've got their own life, and they are with them because they want to be.”

(Manager M9)

### 3.4 Finalised typology

Depending on subcategories within these categories, a total of twelve mentoring models were identified. As a second step to the development of the final typology, these mentoring models were then grouped into one of two overall categories of programmes.

Mentoring programmes that focussed broadly on helping and supporting young people with their character, personal and social development were allocated to ‘Personal and Developmental’ Mentoring (PDM). Programmes that focussed on helping and supporting the young person with regard to their educational learning, future career choices and employability were allocated to ‘Academic and Employability Mentoring’ (AEM). PDM and AEM can be seen as broader mentoring groupings, each encompassing six mentoring models.

**Fig. 2:**
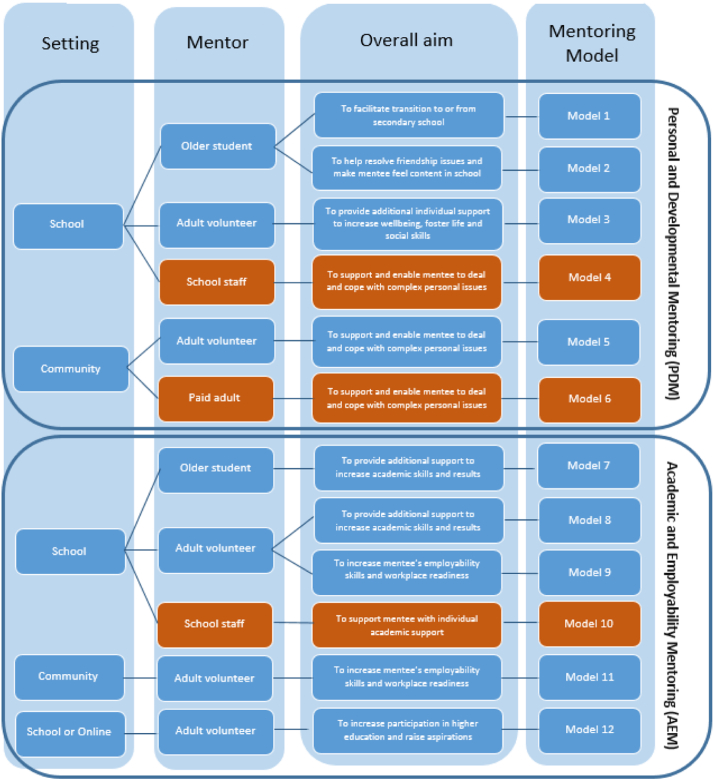
Typology of formal mentoring programmes for young people in secondary schools in the UK. Note: This typology does not allow for any inferences made regarding the model's (cost) effectiveness or efficacy. The representativeness of the models might differ between different countries of the United Kingdom. An accompanying guidance note is available from authors. Personal and Developmental Mentoring (PDM) = Mentoring programmes focussed on helping and supporting young people with regard to their character, personal or social development, life skills, building resilience or to help with current experiences in school and beyond. Academic and Employability Mentoring (AEM) = Mentoring programmes focussed on helping and supporting young people with regard to their educational learning or academic progress in school, future career choices and ambitions and employability skills. Colour-coding: Some participants questioned whether these orange-coloured programmes could be considered as mentoring programmes due to the type of mentor utilised in those programmes. The aim of this classification is to represent all programmes that are seen or described as mentoring programmes, thus these models are still included in the classification.

In total, 15 of the 28 formal mentoring programmes fitted exclusively into PDM, whereas 13 fitted in AEM. The final typology is presented in Fig. 2. A description of the twelve models is found in Table 2. A guidance note to the typology is available on request from the corresponding author.

**Table 2:** Description of the twelve mentoring models.

	Aim category	Type of mentee	More specific aim	Type of mentor	Setting	Format	Method of communication	Activities	Timing	Intensity/Frequency	Overall length	Extent of supervision	Delivery organisations and funding
Model 1	PDM	Individual or group of young people in year 7 or Year 10	To facilitate transition to or from secondary school	Older student	School	One-to-one or group	Face-to-face	Talking, class-room based activities	Within school time	Weekly or biweekly short sessions	Few months to 1year	Supervision by school coordinator	Delivered by schools through internal funding
Model 2	PDM	Individuals struggling with friendship issues	To help resolve friendship issues and feeling content in school	Older student	School	One-to-one	Face-to-face	Talking, class-room based activities	Within school time	Weekly or biweekly short sessions	Few weeks or couple of months, flexible	Supervision by school coordinator	Delivered by schools through internal funding
Model 3	PDM	Individual or group of young people struggling with overall confidence or low-level personal issues	To provide additional individual support to increase wellbeing, foster life and social skills	Adult volunteer	School	One-to-one or group	Face-to-face	Talking	Within school time	Frequent 30–60 minute sessions	Flexible in length depending on need	Supervision by organising coordinator	Organised either through schools themselves through internal funding or from mentoring organisations and external funding
Model 4	PDM	Individual with medium or high level of complex personal issues, needs and vulnerabilities	To support and enable mentee to deal and cope with complex personal issues	School staff	School	One-to-one	Face-to-face	Talking, class-room based activities	Within school time	Frequent short sessions	Flexible in length depending on need	Supervision not certain	Delivered by schools through internal funding
Model 5	PDM	Individual with medium or high level of complex personal issues, needs and vulnerabilities	To support and enable mentee to deal and cope with complex personal issues	Adult volunteer	Community	One-to-one	Face-to-face	Activity-based, outside of class-room	Outside of school time	Weekly, one or two hour-long sessions	5 months +, some flexible	Supervision by coordinator	Delivered by mentoring and youth organisations through external funding
Model 6	PDM	Individual with medium or high level of complex personal issues, needs and vulnerabilities	To support and enable mentee to deal and cope with complex personal issues	Paid adult	Community	One-to-one	Face-to-face or team of mentors	Talking, activity-based	Within or outside school time	Hourly sessions multiple times per week	Few weeks, flexible	Team supervision	Delivered by mentoring and youth organisations through external funding
Model 7	AEM	Individual or group of young people seen to struggle with current learning and academic work	To provide additional support to increase academic skills and results	Older student	School	One-to-one or group	Face-to-face	Talking, class-room based activities	In or after school time	Half an hour, weekly sessions	Few weeks, flexible	Supervision by school coordinator	Delivered by schools through internal funding
Model 8	AEM	Individual or group of young people seen to struggle with current learning and academic work	To provide additional support to increase academic skills and results	Adult volunteer	School	One-to-one or group	Face-to-face	Talking, class-room based activities	In or after school time	Hourly sessions every few weeks, some weekly	Few weeks up to few months	Supervision by organising coordinator	Organised either through schools themselves through internal funding or from mentoring organisations and external funding
Model 9	AEM	Individual or group of young people seen to struggle with choosing right career and planning next steps	To increase ambitions, employability skills and workplace readiness	Adult volunteer	School (or sometimes Workplaces)	One-to-one or group	Face-to-face	Talking, class-room based activities, sometimes including work visits	In or after school time	Hourly sessions every few weeks, some weekly	Few weeks up to few months	Supervision by coordinator	Organised either through schools themselves through internal funding or from mentoring organisations and external funding
Model 10	AEM	Individual young person identified to be at current risk of dropping out of school	To increase likelihood that mentee stay in school	School staff	School	One-to-one	Face-to-face	Talking	In school time	Frequent short sessions	Few weeks, as needed	Supervision not certain	Delivered by schools through internal funding
Model 11	AEM	Individual young person identified to be at high risk of not staying in education, employment or training	To increase likelihood of staying in education, employment or training long-term	Adult volunteer	Community	One-to-one	Face-to-face	Activity-based, sometimes including work placement	In or after school time	Hourly sessions every week or bi-weekly	Few months up to few years	Supervision by programme coordinator	Delivered by mentoring and youth organisations through external funding
Model 12	AEM	Individual or group of disadvantaged young person seen to not be likely to proceed to higher education	To increase participation in higher education and raise aspirations	Adult volunteer (predominantly University students)	Online or school	One-to-one or group	Face-to-face or online	Talking, class-room based activities, can include university visits	In or after school time	Half an hour, every few weeks	Few weeks or months	Supervision by coordinators	Delivered by universities or partnership federations through internal funding

Note: Please note that this refers to a general and overall description of these different models based on the programmes that have been discussed in interviews. Individual programmes fitting under one models might still differ in some details from what is presented. PDM = Personal and Developmental Mentoring. AEM = Academic and Employability Mentoring.

### 3.5 Consultation process

As outlined in Fig. 1, the consultation process had six sequential steps, involving feedback from programme managers and experts. Participants were generally able to follow the way programmes were classified in the typology. They regarded the typology as comprehensive and capable of including the mentoring programmes that they were familiar with.

“When I look at the granular models that you provide, it's definitely comprehensive. I don't see that there is anything missing”

(Expert E3)

However, it became clear that participants differed in their conceptualisation of mentoring and what was considered to be a mentoring programme. Overlaps of some models with befriending, counselling and coaching were drawn.

“The major question mark I have over the classification is, are all of the schemes actually mentoring schemes or are they just calling themselves mentoring schemes?”

(Expert E1)

“For me, some of them [mentoring models] are a mix of befriending and coaching […], others are more mentoring approaches I would recognise”

(Expert E1)

Moreover, some participants did not see programmes with paid adults as mentoring programmes.

“And I would ask the question, is a paid professional a mentor, or is it a paid professional doing some support?”

(Expert E2)

Consequently, colour-coding had been included in the typology to include this feedback. Another expert participant indicated that mentoring programmes that only entail a few sessions were not regarded as mentoring.

“There is no way I would think of a couple of sessions as mentoring. That's not mentoring.”

(Expert E4)

Most of the participants felt that the typology was representative of programmes that they were aware of. Two participants highlighted that they felt that mentoring programmes cannot be classified.

“In our experience, mentoring schemes don't fit into boxes this neatly.”

(M8, written feedback)

Participants viewed the typology as a useful aid in navigating and understanding different programmes. They also alluded that the typology could help practitioners to describe their programmes which in turn should aid the programmes' evaluation.

“I do believe that we do need these models, we do need to be more classified, in order to be able to evaluate our work better.”

(Expert E3)

“The classification I think will be very helpful […] in helping people to understand that […] in setting up mentoring and delivering mentoring, that having a clear understanding and looking at the different classifications, will help the implementation of a better run project. Which you can then, measure.”

(Expert E2)

“I think the classification is, as indicated earlier, very useful in helping […] myself and my team to better reflect on and evaluate what we are delivering in our context.”

(M14, written feedback)

Furthermore, participants appreciated that the typology itself was not prescriptive of what mentoring should be.

“You are not being overly prescriptive. […] you are not […] dictating what mentoring should be or should not be, and that context, which is good.”

(Expert E4)

The typology was also perceived to act as a starting point for more work in this field and to aid facilitation between organisations that follow similar mentoring models. Appendix D details the main feedback and changes made to the typology at the different stages of the consultation process.

## 4 Discussion

Formal mentoring programmes for young people in UK secondary schools can be classified into twelve distinct mentoring models within two broad groupings of ‘personal and developmental’ and ‘academic and employability’ mentoring programmes. The typology derived from the study differentiates programmes according by three overarching categories: a programme's setting, type of mentor and programmes' overall aim. This study found that formal youth mentoring programmes are heterogeneous and that differences exist in the way ‘mentoring’ is conceptualised.

This is the first typology that has been created for the classification of formal mentoring programmes for young people in secondary schools in the UK. Contrary to other typologies (Philip & Hendry, 1996; Philip & Spratt, 2007; Simon & Eby, 2003), this typology presents a way to distinguish between formal mentoring programmes that are in existence, hence allowing insight into the types of programmes for a specific target group.

The methodology used to derive the typology differs from previous attempts (Philip & Spratt, 2007; Simon & Eby, 2003; Sipe & Rogers, 1999) and ensures that the typology is grounded in participants' descriptions of programmes. This typology differs from previous typologies in the UK (Ford, 1998; Philip & Hendry, 1996; Philip & Spratt, 2007) in that it allows each programme, despite variation in their format, delivery and a range of other characteristics, to be allocated to one and only one mentoring model. Consistent with previous studies (Mass Mentoring Partnership, 2007; Sipe & Rogers, 1999), this study revealed that a total of 33 different categories can be used to differentiate between existing programmes, spanning across programme structures and formats, detailed information on mentees and mentors involved and organizational details and practices. This acknowledges the diverse ways in which mentoring programmes within one mentoring model operate and deliver their programme. The categories derived through this work are complementary to previously published materials, such as Dawson's 16 design elements for models of mentoring in a higher education contexts (Dawson, 2014). In line with Dawson's (2014) conclusions, we believe that these categories can help practitioners and researchers to clearly describe their programme, be explicit about what is meant by ‘mentoring’ and can provide points for discussions for those who design mentoring programmes. Not specifying the details of a mentoring programme undermines understanding and can hinder evaluations of the effectiveness of different types of mentoring programmes.

The main distinction used within our typology, the overall aim of a programme, is similar to Karcher's work which distinguished what was referred to as ‘instrumental’ and ‘developmental’ mentoring (Karcher et al., 2006). This indicates that mentoring programmes for young people in the UK, similar to the US, have generally been applied for young people in the context of helping young people work towards beneficial educational and employment outcomes or to help young people with their personal and social development. Philip and Henry also differentiated mentoring programmes by their overall aim (Philip & Hendry, 1996).

The consultation process revealed that some participants did not perceive all mentoring models to be ‘mentoring’ which was the main reason for some models to be colour-coded. Participants drew links between mentoring, befriending and coaching. The difficulty of defining what ‘mentoring’ is and consists of is in line with the previous literature (Stewart & Openshaw, 2014). Moreover, some participants voiced that they did not regard paid mentors as mentoring due to their potential role conflict and the voluntary nature of mentoring being a key factor in why mentoring is seen to be successful.

It was further recognised by participants that mentoring programmes might aim to achieve multiple aims, crossing the boundaries between a focus on academic and employability to make long-lasting change in other domains of life as well. We acknowledge that programmes might aim to impact on both, directly and indirectly, and that the aim of the relationship between mentor and mentee might change over time, depending on the specific needs of the young person taking part. For example, whereas the mentoring relationship might focus on a more practical-oriented task in the beginning, through the establishment of a trusting relationship, this might shift and might then focus on sharing personal information.

Developing the typology allowed us to learn about common programme practices of mentoring programmes within the UK. Most programmes were delivered on a one-to-one, face-to-face basis however there were also programmes using group mentoring and online mentoring which have been referred to as newer models of youth mentoring in the literature. Moreover, some programmes combined mentoring with other activities as has also been reported from programmes available in the USA (Deutsch, Wiggins, Henneberger, & Lawrence, 2013). It further became clear that most programmes entailed eligibility criteria for young people to access the programme instead of programmes being available for all young people. Evaluations of programmes have been shown to be more effective when involving youth with some or a moderate level of risk factors or difficulties present, compared to those presenting with high or no risks (DuBois et al., 2011; Schwartz, Rhodes, Chan, & Herrera, 2011).

Looking in detail at programmes models available in the United Kingdom highlights that some contain features which would seem to be at variance with what evidence has suggested is best. For instance, research has reported that mentoring carried the greatest impact for those formal relationships that last one year or longer(Grossman & Rhodes, 2002), yet the majority of programmes included in this study reported a length of less than a year, with only one programme being longer than twelve months. This is particularly concerning, as those matches that are less than a year or those that terminate early or prematurely have been associated with potential harmful effects for youth (Grossman & Rhodes, 2002; Spencer, 2007; Spencer et al., 2017).

### 4.1 Strengths and limitations

Due to the eligibility criteria and sampling framework, the generalisability and representativeness of our study is limited to mentoring programmes within the UK. Despite a pilot search undertaken confirming that most mentoring organisations had a web-presence, the search was not exhaustive and might not have included smaller programmes or recently set up programmes. Moreover, another limitation of the research is that characteristics of non-participations at each stage of the non-participation were not captured. All mentoring programmes described in interviews were able to be assigned to one and only one of the twelve mentoring models but this does not mean that the models are equally common and that the number of models is representative of all UK programmes. Further, in cases where multiple programmes were provided by participants, some interviews did not allow thorough coverage of each programme, thereby limiting the typology to the description of the programmes that were given in interviews. Compared to other countries of the UK, programmes based in England had a proportionally higher representation indicating that the mentoring models established in England might not be available in the other countries of the UK. Although the typology was seen by experts and programme managers to be representative, there are no objective data available which could be used to confirm or refute this. The typology does not allow for any inferences made regarding each model's (cost-) effectiveness or efficacy.

However, involving programme managers to establish the typology was not only best suited to the needs of the research but also involved a novel approach. The use of maximum variation sampling and three sampling waves allowed for a range of programmes to be selected thereby increasing the generalisability and representativeness of the typology. A consultation process was implemented to allow participants to help improve and validate what was found which has not been done in past studies. Reliability of data was ensured by following the same research principles in each interview and all audio-recordings being transcribed by the same researcher.

### 4.2 Implications and future research

This research emphasises the heterogeneity of programmes and what is understood to be mentoring, emphasising (i) the need for mentoring organisations to thoroughly describe what their mentoring programme(s) entail and (ii) the need for a definition of mentoring that takes into account current developments in mentoring, such as mentors being paid (Eddy et al., 2017). The different categories by which to distinguish between programmes that were brought up in this work can provide guidance on what information should be covered within the mentoring programme's description. The typology can also be used to classify programmes into groups which can help inform subgroup analysis of different mentoring models when synthesising the available evidence on their effectiveness such as in future systematic reviews of the literature.

Whereas many different programmes are typically grouped together in existing systematic reviews, synthesising the evidence of mentoring programmes based on grouping them by their different mentoring models can provide a more useful approach to this. The typology itself can help practitioners and researchers to help allocate their programmes into one of the mentoring models. Doing so can help practitioners to identify similar mentoring programmes. The typology is useful to researchers as it provides guidance about the types of programmes in existence which can help when seeking to evaluate ‘mentoring’ as an intervention. This ultimately paves the way for a more systematic evaluation of the effectiveness of mentoring programmes as interventions for young people. Future research is required to test the typology's generalisability within the UK and internationally. This might be achieved by surveying existing mentoring programmes which will also shed light on the prevalence of each mentoring model. Given the small number of online mentoring programmes or programmes using group mentoring, further research is needed into these newer models of mentoring, their prevalence and process of change. Another distinction that has not been brought up in our work but that has been made in the mentoring literature is between homogenous and heterogeneous groupings of mentor and mentee (Darling, Bogat, Cavell, Murphy, & Sánchez, 2006). It would be important to find out how far mentor characteristics match those of mentees and whether the type of grouping can impact on the outcomes of mentoring programmes. The need for a wide-ranging and robust evaluation of mentoring programmes in the United Kingdom persists. Particularly in times where resources are limited, it will be important to assess particularly the cost-effectiveness of different mentoring programmes; meaning that future evaluations should incorporate cost-effectiveness calculations. A multi-programme evaluation might possibly need be considered for the design of such a study. Given the various programmes in existence, it will be further of importance to study programme-level moderators of effectiveness (Karcher & Nakkula, 2010).

Given the importance of the length of relationship and quality of relationship on the outcomes of mentoring that has been alluded to in the mentoring literature (Rhodes, Schwartz, Willis, & Wu, 2017), it would be of further importance to investigate the potential of different mentoring models in achieving certain types of mentoring relationships, as mentioned in Karcher and Nakkula's typology (Karcher & Nakkula, 2010). Related to this aspect and given the uncertainty about whether paid mentors should be seen as mentors, it would be important for future research to look at how different types of mentors can impact on the relationship that is being formed between mentor and mentee and how this subsequently can impact on the outcomes of a programme. Similarly, it would be of importance to look at other programme factors than can impact on the effectiveness of programmes. Based upon a review of the empirical and practice literature, a group of researchers developed the ‘Elements of Effective Practice for Mentoring’, currently in its fourth edition, which described identified programme practices that were associated with beneficial outcomes (Garringer, Kupersmidt, Rhodes, Stelter, & Tai, 2015). It is not yet known in how far, if at all, these practices are followed within the different mentoring models in the United Kingdom. Looking closer into the scope of different mentoring models and their practices will help to establish the potential effectiveness of different models. Given that we acknowledge that mentoring programmes, particularly longer-term programmes, might change their focus over time, it would be important to assess which mentoring models are able to do this, how they achieve this and how this might change the mentoring relationship. To understand the challenges and situation of each model, a better understanding of the context surrounding the delivery, development and maintenance of mentoring programmes is required.

## 5 Conclusions

Although mentoring programmes are heterogeneous in the ways that they are delivered, it is possible to identify key characteristics and distinguish between different mentoring models based on their overall aim, setting and the type of mentor used. This work emphasises the need for formal mentoring programmes to be well defined and described. The typological approach to mentoring programmes helps academics and service providers understand what is being delivered, for whom, and how, which is a necessary precursor to any evaluation, service design or commissioning process.

## Funding

The work was undertaken with the support of The Centre for the Development and Evaluation of Complex Interventions for Public Health Improvement (DECIPHer), a UKCRC Public Health Research Centre of Excellence. Joint funding (MR/KO232331/1) from the British Heart Foundation, Cancer Research UK, Economic and Social Research Council, Medical Research Council, the Welsh Government and the Wellcome Trust, under the auspices of the UK Clinical Research Collaboration, is gratefully acknowledged.

## Conflict of interests

None.

## Appendix A Supplementary data

Supplementary material Image 1
