# Supplementary material for: Developing a typology of mentoring programmes for young people attending secondary school in the United Kingdom using qualitative methods
Source: Child Youth Serv Rev. 2018 May;88:401–15. doi: 10.1016/j.childyouth.2018.03.025 (PMC5946908; doi:10.1016/j.childyouth.2018.03.025)
Supplement: Supplementary file 1 — Supplementary material [file mmc1.docx]

# Supplementary Material

## Appendix A Description and results from website search

| Name of search website | Description of search website | Search terms used | Number of search results to screen for eligibility | Number of eligible organisations (duplicates removed) |
| --- | --- | --- | --- | --- |
| Mentoring and Befriending Foundation (MBF) website search (Available at <http://www.mandbf.org/>. Accesssed August, 9^th^ 2015 ) | The MBF operates a database of mentoring programmes operating in the UK, predominantly in England. | n/a | 465 | 123 |
| Scottish Mentoring Network (SMN) website search  (Available at: <http://scottishmentoringnetwork.co.uk/>. Accessed August, 10^th^ 2015) | This website entails a database of mentoring programmes operating in Scottland. | n/a | 166 | 15 |
| England and Wales Charity commission website  (Availble at: <https://www.gov.uk/government/organisations/charity-commission>. Accessed August, 11^th^ 2015) | This website contains a searchable register of all registered charities within England and Wales. | ‘mentor’ match any word, charity name;  ‘mentoring’, match any word, charity name;  ‘school-based mentoring’, match all words, charity activities | 31 | 3 |
| Scottish Charity Regulator Website (Availabel at: <http://www.oscr.org.uk/>. Accessed August, 11^th^ 2015) | This websites contains a searchable register of all registered charities within Scotland. | ‘mentor’ and ‘mentoring’ | 40 | 1 |
| Charity Commission for Northern Ireland Website (Available at: <https://www.charitycommissionni.org.uk/>. Accessed August, 11^th^ 2015) | This website contains a searchable register of all registered charities within Northern Ireland. | ‘mentor’ match any word, charity name;  ‘mentor’ match all words, charity name; ‘mentoring’, match any word, charity name; | 71 | 1 |
| Welsh Assembly Government (Available at: [www.wales.gov.uk](http://www.wales.gov.uk). Accessed August, 12^th^ 2015) | This website contains further information about organisations in Wales. | ‘mentor’, ‘Mentoring’, ‘peer mentoring’, ‘student mentoring’ | 8 | 1 |
| Google search Cardiff (Available at: [www.google.co.uk](http://www.google.co.uk). Accessed August 12^th^ 2015) | Google is an online web search engine; using a range of terms. | ‘school mentoring programme’, ‘school mentor programme’ and ‘mentoring programme secondary school’ in addition to ‘Cardiff’; first three pages looked at | 22 | 5 |
| Google search Belfast (Available at: www.google.co.uk. Accessed August 12th 2015) | Google is an online web search engine; using a range of terms. | ‘school mentoring programme’, ‘school mentor programme’ and ‘mentoring programme secondary school’ in addition to ‘Belfast’; first three pages looked at | 12 | 5 |
| Additional organisations found through other websites and links |  |  |  | 8 |
| TOTAL | |  | 815 | 163 |

## Appendix B Overview of all categories to distinguish between programmes

| **Category domain** | **Categories** | **Category options** | | | | | |
| --- | --- | --- | --- | --- | --- | --- | --- |
| **Overall aim** | **Aim of mentoring programme^a^** | To help with personal skill and character development, to help reach their potential | To help with widening participation in higher education and raise awareness | To help young people with learning and educational experiences in school | To help young people feel happier and with mental health needs | To help young people with employability skills and workplace readiness | To help young people with transition into or out of secondary school |
|  | **Main focus of prevention^a^** | Primary prevention | Secondary prevention | Tertiary prevention |  | | |
| **Mentor description** | **Type of mentor^a^** | Adult volunteers (incl. university students) | Paid adults | Older school students/  peers | School staff members | Combi-nation of mentors |  |
|  | **Remuneration of mentor^a^** | Not paid | Paid for expenses only (travel and meeting costs) | Paid a subsistence for duration of mentoring in addition to cover travel and meeting costs) | Paid a salary |  | |
| **Mentee description** | **Type of mentee^a^ (*multiple options may apply*)** | Whole cohort/year group within school | Year 7 students | Young people of disadvantaged backgrounds | Young people presenting with difficulties at school/disengaged | Young person with social or emotional difficulties | Anyone of secondary school age |
|  | **Targeted or universal programme** | Targeted | Universal |  | | | |
|  | **Voluntary participation from young person^a^** | Yes | No |  | | | |
| **Relationship details** | **Peer or adult-young person mentoring^a^** | Peer-to-young person | Adult-to-young person |  | | | |
|  | **Mentor’s role in young person’s life** | Embedded to some degree in life of young person next to mentoring programme (i.e. present in school) | Present only for mentoring programme |  | | | |
|  | **Formal matching process** | Yes | No |  | | | |
|  | **Number of mentors working with young person** | One | Two | Three or more |  | | |
|  | **Can mentor and mentee continue to work together after end of formal mentoring programme?** | Yes | No |  | | | |
| **Details of delivery of mentoring programme** | **Predominant setting^a^** | School | Community | Online |  | | |
|  | **Method of delivery^a^** | Face-to-face only | Online only | Combination of face-to-face and online |  | | |
|  | **Format^a^** | One-to-one mentoring | Group mentoring | Combination of group and one-to-one mentoring |  | | |
|  | **Time of delivery (*Multiple options may apply*)** | In school time but during lunch break or registration | In school time and during lesson | In school but after school | Outside of school time | Combination or in and outside of school time |  |
|  | **Intensity** | Below or half an hour | Half an hour to an hour | One hour up to two hours | Two or more hours | Variable |  |
|  | **Frequency^a^** | Multiple times per week | Weekly | Bi-weekly | Once a months | Less than once a months |  |
|  | **Overall duration^a^** | Under or three months | Three to six months | Six to twelve months | 1-2 years | More than two years | Variable |
|  | **Is there an option for young people to extent mentoring programme?** | Yes | No |  | | | |
| **Details of mentoring session** | **Type of activities *(Multiple options may apply)*** | Talking or chatting | Activity-or interest-based (i.e. sports, arts and crafts) | School-work focussed (i.e. homework, learning and revision strategies) | Employability activities (CV writing, applications, interview preparation) | Future career activities and career planning | Other |
|  | **Provision of mentoring on own or with other activities^a^** | On its own | With other activities as part of programme (i.e. work visits, placements, career talks, community projects) |  | | | |
|  | **Who is present in mentoring session?** | Mentor and mentee only | Mentor, mentee and programme staff member | Mentor, mentee and staff member with supervisory function | Mentor, mentee and family members | Other |  |
|  | **Who generally determines content of activities and sessions?** | Mentor | Mentee | Both mentor and mentee | Programme staff | None |  |
|  | **Structured vs unstructured sessions^a^** | Yes | No |  | | | |
| **Organisational details** | **Initiator organisation** | Secondary school | University | Youth organisation | Mentoring organisation | Partnership federation |  |
|  | **Provider organisation^a^** | Secondary school | University | Youth organisation | Mentoring organisation | Partnership federation |  |
|  | **Number of mentees/year^a^** | 0-10 | 10-20 | 20-50 | 50-100 | 100-500 | More than 500 |
|  | **Geographic coverage^a^** | Single city | Multiple cities | Region | Multiple regions | Nationwide |  |
|  | **Country** | England | Scotland | Northern Ireland | Wales | More than one country |  |
|  | **Funding^a^** | Internally funded | Externally funded |  | | | |
|  | **APS accreditation^a,b^** | Yes | No |  | | | |
|  | **Theoretical stance of organisation^a^** | Defined theoretical stance | No theoretical stance |  | | | |

## Appendix C Short description of categories derived from participants or analyses

| **Categories derived from participants** | | |
| --- | --- | --- |
| **Category** | **Short description of category** | **Number of participants mentioning this category (%)^a^** |
| Aim of mentoring programme | Purpose, or the aim and objectives and the focus of the mentoring scheme, i.e. whether this was focussed on educational attainment and outcomes, widening participation at university or at helping the young person with their personal development. | 14 (61%) |
| Type of mentee | Characteristics of the mentees i.e. whether or not the mentee had to meet certain referral criteria in order to take part, the kind of referral criteria or the age of the mentee. | 11 (48%) |
| Overall duration | Whether mentoring was provided for a few weeks, a few months or year(s). | 8 (35% |
| Structured vs. unstructured sessions | Whether or not mentoring programmes followed a specific pre-defined and agreed programme or session plans. | 7 (30%) |
| Predominant setting | The predominant setting in which the mentoring session took place, i.e. in the school, in the wider community/at workplaces, or online. | 7 (30%) |
| Method of delivery | How the mentoring session was delivered, i.e. whether this was face-to-face, online or a combination of the two. | 7 (30%) |
| Type of mentor | Characteristics of the mentor, i.e. whether this individual was from an external organisation, works within the school, the age of the mentor and other characteristics. | 6 (26%) |
| Peer or adult-to-young person mentoring | Whether the mentoring programme followed the model of a peer mentoring programme, where young people mentor other young people, or whether an adult mentors a young person. | 6 (26%) |
| Remuneration of mentor | Whether mentors received a salary to deliver the mentoring or whether they acted as volunteers. | 5 (22%) |
| Format | Whether mentoring was provided for a group of young people or whether it was provided on an individual, one-to-one, basis. | 5 (22%) |
| Provider organisation | Organisation that is responsible for the delivery of the mentoring programme, i.e. whether this was provided as part of school, by an external mentoring organisation, by a university or a workplace. | 4 (17%) |
| Voluntary participation from young person | Whether or not young person was voluntarily taking part in programme. | 4 (17%) |
| Provision of mentoring on own or with other components | Whether the mentoring programme was solely focussed on mentoring or entailed mentoring as one component in combination with other components such as such as university visits, a sport activity, or other types of intervention. | 4 (17%) |
| APS accreditation | Whether or not the mentoring programme was awarded the Approved Provider Standard (APS), a quality standard developed and provided by the Mentoring and Befriending Organisation. | 2 (9%) |
| Funding | Whether the mentoring programme was externally or internally funded. | 2 (9%) |
| Frequency | Frequency (i.e. weekly, monthly) of the mentoring provision. | 1 (4%) |
| Theoretical stance of organisation | Whether the organisation had a defined theoretical stance underpinning the mentoring programme. | 1 (4%) |
| Main focus of prevention | Whether programme was aimed at preventing a negative outcome, such as exclusion from school (primary prevention) or whether the programmes aimed to help those that have already presented with risk factors for a negative outcome (secondary prevention). | 1 (4%) |
| Geographic coverage | Whether this was provided in a rural or urban area. | 1 (4%) |
| Size of organisation | How many young people took part in the programmes across the organisation. | 1 (4%) |
| **Categories derived through data analyses** | | |
| Targeted or universal programme | Whether or not the programme was targeted to some young people or whether this was universal. | |
| Mentor’s role in young person’s life | Whether or not the mentor was present in the young person’s life outside of the mentoring programme. | |
|  |  |  |
| Format matching process | Whether or not a formal matching process was undertaken between mentor and mentee. | |
| Number of mentors working with young person | Number of mentors that were working with the young person as part of the mentoring programme. | |
| Option to extend programme | Whether or not young people were able to extend the mentoring programme at the end of the initial programme. | |
| Context after end of formal mentoring programme | Whether or not mentors and mentees were allowed and supported to continue working together (e.g. meeting, through texting) and staying in contact at the end of the formal mentoring programmes. | |
| Time of delivery | Time of delivery, such as whether the mentoring programme took place in school time, during breaks, after school or outside of school time. | |
| Intensity | Actual length of each mentoring session. | |
| Type of activities | Type(s) of activities that were undertaken as part of the mentoring programme such as whether the mentor and mentee engaged in talking and chatting only or whether this included engagement in an activity such as sports or others. | |
| Who is in session | Type of people present during the mentoring session, i.e. whether this was only the mentor and mentee or whether other people are involved (e.g. coordinator). | |
| Who determines content of activities and sessions | Who generally determined and made decision about the content of the activities and sessions, e.g. whether this was generally young person and/or mentor-led or determined by programme staff. | |
| Country | Country in which the programme operated. | |

## Appendix D Changes made to typology as part of consultation process

| **Feedback from initial interviews with experts** | |
| --- | --- |
| **Summary of feedback/recommendations** | **Actions taken in revision of typology** |
| ***Key feedback points from consultation*** |  |
| Whether mentoring programmes working with paid mentors are mentoring programmes. | As these programmes were still seen by some providers as mentoring programmes, they have been left in the typology but colour-coded to indicate the views of experts. |
| Potentially difficulty for practitioners to allocate their programmes into either PDM or EEM. | Typology has been updated to provide more specific examples of the overall purpose of programmes and target group. |
| Relatively small sample size compared to number of mentoring programmes in existence in UK. | Numbers removed to avoid confusion. It is explicitly stated that the typology is based on a purposive sample of organisations but that prevalence of the different mentoring models in the UK is unknown. |
| ***Specific recommendations*** |  |
| That the age range of participants in programmes form part of typology. | Accepted. Age range is now mentioned in the background description. |
| To explain what definition of mentoring was used to guide the typology. | Accepted. It is explicitly stated that any programme that defined itself as a mentoring programme was included in the typology. |
| To not use the word ‘holistic’ to describe PDM programmes as these could also be described as focussed. | Accepted. The word ‘holistic’ in description of PDM has been removed. |
| To include social outcomes as part of PDM programmes, e.g. increasing young people’s social networks. | Accepted. Social outcomes are mentioned as part of description of PDM. |
| To note that all programmes typically work with a certain target group of young people, mainly young people seen ‘at risk’ or disadvantaged. | Accepted. This has been noted within the overall description. |
| To acknowledge the existence of contextual factors as part of mentoring programmes. | Accepted. Contextual factors are acknowledged in the background description. |
| To include programme structure and processes as categorisations in typology. | Accepted. This has been included in the description of the twelve mentoring models. |
| To include type of mentee as a category within the typology. | Accepted. |
| To include whether a programme is run internally or externally as a category in the typology. | Accepted. This has been included in the description of the twelve mentoring models. |
| To include method of communication as a category in the typology. | Accepted. This has been included in the description of the twelve mentoring models. |
| To include the motivation of a mentor as a category in the typology. | This has not been included as this was not generally mentioned by interviewees. However, this topic is alluded to in the discussion of programmes. |
| **Feedback from written consultation with programme providers** | |
| **Summary of feedback/recommendations** | **Actions taken in revision of typology** |
| ***Key feedback points from consultation*** |  |
| Potential overlap between PDM and EEM; and programmes trying to achieve multiple aims. | The category ‘aim’ was changed to ‘overall aim’, highlighting that programmes are understood to focus on one overall aim, but that they might also focus on multiple other aims. |
| Difficulty in allocating programmes into one and only one mentoring model. | Overarching categories within typology were changed to simplify the process of allocating programmes to models in the order of ‘setting’, ‘type of mentor’, ‘overall aim’, and ‘mentoring model’. Type of mentee was moved to the descriptions of models to make the typology clearer. |
| ***Specific recommendations*** |  |
| To clarify terms ‘aim’ and ‘outcome’ | Accepted. These terms are further explained in the description of the classification. |
| **Feedback from follow-up interviews with experts** | |
| **Summary of feedback/recommendations** | **Actions taken in revision of typology** |
| ***Key feedback points from consultation*** |  |
| To add a ‘guidance’ note to allow interpretation of classification | Accepted. A guidance note has been prepared and is available from the corresponding author on request. |
| ***Specific recommendations*** |  |
| To note that Model 1 includes students transitioning into sixth form | Accepted. The type of mentee has been extended to include students transitioning into sixth form. |
| To note that Model 12 also occurs with adult volunteers, not only university students | Accepted. The type of mentor in this model has been changed to ‘adult volunteer’. In the description of mentors, it is noted that particularly university students work as mentors in this model. |
| To clarify the term ‘supervision’ used in description. | Accepted. |
| To consider changing term ‘educational’ to ‘academic’ | Accepted. The Model EEM has been changed to AEM and the term has been explained within the description of AEM. |
| To consider changing visual representation and design of typology to make it more design-attractive. | This has been considered by the team. |
| To add clarification around term ‘school staff’. | Accepted. Descriptions of the type of mentors have been included in the guidance note. |
| To specify definition of mentoring used in research. | Accepted. This is highlighted in the background section of the classification. |
